# Economic evaluation and analyses of hospital-based electronic medical records (EMRs): a scoping review of international literature

**DOI:** 10.1038/s41746-022-00565-1

**Published:** 2022-03-08

**Authors:** Kim-Huong Nguyen, Chad Wright, Digby Simpson, Leanna Woods, Tracy Comans, Clair Sullivan

**Affiliations:** 1 Centre for Health Services Research, Faculty of Medicine, The University of Queensland, Brisbane, QLD Australia; 2 Global Brain Health Institute, Trinity College Dublin, Dublin, Ireland; 3 Australasian Kidney Trials Network, Faculty of Medicine, The University of Queensland, Brisbane, QLD Australia; 4 Clinical Informatics Director (Research), Metro North HHS, Queensland Health, Herston, QLD Australia

**Keywords:** Health care economics, Economics

## Abstract

Digital transformation is expensive and rarely smooth, often leading to higher costs than anticipated. It is challenging to demonstrate the contribution of digital health investment in achieving the healthcare aims of population health and workforce sustainability. We conducted a scoping review to understand how electronic medical record (EMR) implementations in the hospital setting have been evaluated using cost–benefit analysis (CBA) approaches. The review search resulted in 1184 unique articles, a final list of 28 was collated of which 20 were US-based studies. All studies were published in 2010–2019, with fewer studies published in more recent years. The data used to estimate benefits and costs were dated from 1996 to 2016, with most data from 2000 to 2010. Only three studies were qualified as using cost–benefit analysis approaches. While studies indicated that there is a positive impact from the EMR implementation, the impacts measured varied greatly. We concluded that the current literature demonstrates a lack of appropriate and comprehensive economic frameworks to understand the value of digital hospital implementations. Additionally, most studies failed to align fully to the quadruple aims of healthcare: they focused either on cost savings and/or improved patient outcomes and population health, none investigated healthcare-workforce sustainability.

## Introduction

The electronic medical record (EMR) industry is expanding rapidly, growing from nearly nonexistent in 2000 to being worth more than $31 billion a year in 2018^1^. Globally, EMR adoption has been recognized as an important step toward healthcare modernization—necessary to improve patient outcomes, increase health system efficiency, and unlock extensive research potential in healthcare. For instance, the United States allocated $27 billion for hospitals demonstrating “meaningful usage” of EMR in 2009^2^. EMR adoption is heavily dependent on each country’s socioeconomic policies, health-system organization and financing, and the integration of primary and acute care networks. While many high-income and upper–middle-income countries have reported varying degrees of EMR adoption, lower socioeconomic countries report drastically lower adoption rates, often citing lack of funding and inadequate existing infrastructure as the main obstacles^3^.

The impacts of EMR implementation vary but can be profound for hospital business^4^. These can include improvements in quality, safety, information integrity and completeness, ability to use the data collected in the EMR as part of routine care, and for data analytics to drive quality improvements and research activities. The majority of these impacts, while providing significant value, do not provide immediate financial returns. They are also difficult to quantify and value, and so are usually excluded from traditional, financial-based business cases for EMR investment.

Recently, Lau and Kuziemsky^5^ attempted to synthesize the research and empirical evidence over the past two decades to provide some guidelines to identify and measure the benefits of digital health implementation. The authors stated that the framework presented in the handbook aims to *“provide a high-level conceptual scheme to guide eHealth evaluation efforts to be undertaken by the respective jurisdictions and investment programs in Canada.”* The framework relies heavily on measuring the successful implementation of Information and Communications Technology (ICT) in different settings, a systematic review on the determinants of success in inpatient clinical information systems, and the synthesis of results from health-information system (HIS) evaluations. The three eHealth benefit domains identified in this framework include care quality, access (to care), and productivity. “Care quality” covers a wide range of categories and measures, including patient safety, appropriateness and effectiveness, and health outcomes. “Access” captures the ability of patients and providers to access services, and participation by patients and carers. “Productivity” includes efficiency, care coordination, and net cost.

The guidelines briefly discussed economic methods that are potentially applicable for eHealth benefit evaluation (Chapters 5 and 14 in^5^). These methods were informed by a scoping review of 33 economic evaluation studies applied for HIS, published between 2000 and 2013^6^. A wide range of scopes and types of HIS were considered in this study, ranging from sector systems (e.g., health information-exchange (HIE) networks, primary care EMRs, and immunization information systems) to organization (institutional information systems) and goal-specific systems (computerized provider order entry (CPOE), medication management, disease management, and clinical documentation systems). While informative, the wide and extremely heterogeneous scope provided limited practical guidance for an economic evaluation. EMR applications in primary care settings often have very different targets, and thus short- versus long-term impacts, compared to those in hospital settings. Additionally, the benefits of HIS do not entirely overlap with those generated from EMR for digital hospital implementations.

Owing to the perceived benefits and the “infrastructure” nature of EMR, governments have generally stepped up to provide incentives and subsidies for EMR adoption. However, the cost-effectiveness of this investment has been questioned due to mixed evidence of cost requirements and observed impacts^7^. This is due partly to many providers making only token attempts to satisfy the criteria for meaningful use without capturing the economic values of EMR investments. Additionally, the diversity of EMR implementations in the acute and primary settings complicates the assessment of economic impacts and fiscal significance. Increasingly, resource and fiscal constraints require both the public and private sectors to consider the benefit–cost balance of EMR investments versus other demands, both in and outside healthcare. For large-scale and medium-to-long-term investments and rollouts of EMR to be evaluated properly, robust conceptual frameworks that systematically identify and measure the economic impacts of EMR, backed by healthcare funding agencies, decision-makers, and service providers, are urgently needed.

Our project aims to contribute to this effort by investigating the available options, both in the academic literature and practice, to evaluate the economic values of EMR, and develop a cost–benefit analysis framework to identify, measure and value the impacts of EMR in the Australian hospital-care setting. This paper presents the first piece of work: a comprehensive scoping review examining how EMR implementations in the hospital setting have been evaluated using cost–benefit analysis approaches internationally.

## Methods

**Fig. 1:**
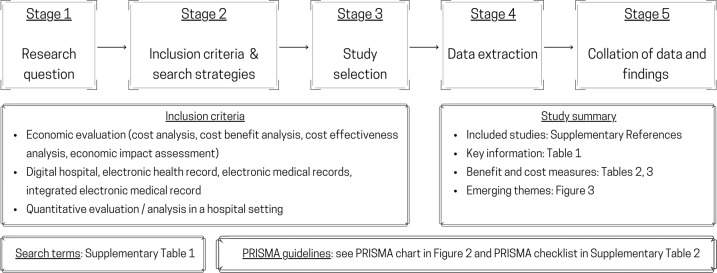
The process of conducting the scoping review.

A scoping review was conducted, following the methodology outlined by Arksey and O’Malley^8^ and Levac et al.^9^. While the main study aims to understand the scope of the literature on CBA of EMR in hospital settings, we were also interested in exploring themes that naturally emerged from the literature. The scoping review was conducted in five stages, as mapped in Fig. 1.

This review method was chosen, instead of a systematic literature review and meta-analysis, because we did not attempt to critically appraise or synthesize quantitative evidence even though CBA studies often reported quantitative results^10^. We propose that both the qualitative and quantitative results from CBA studies in the literature were not necessarily comparable or synthesisable. Those studies might be conducted in different countries, and/or the EMR implementation objectives and practices differ significantly. For theme exploration, a scoping review therefore is the most appropriate starting point, and a base for further systematic reviews with specific quantitative focuses.

### Stage 1—research questions

We reviewed both peer-reviewed and gray literature to understand the evaluation methods, economic benefits and costs of EMR implementation in hospital settings. EMR systems in primary care or nonhospital settings were excluded. More specifically, we asked:

- Q1: How many studies can be classified as a (full) CBA, as opposed to a partial economic analysis? (*That is, studies reporting both benefits and costs, not just benefits or costs only*.) Did those studies clearly discuss benefits and costs incurred by different stakeholder groups, and the analytical perspectives?
- Q2: What were the benefit or cost items? Were they financial or economic? How were they measured and valued? What were the main assumptions or findings on the changes over time of those benefits or cost items, and across settings?
- Q3: What were the main findings regarding the net impacts (i.e., net benefits or net costs)? Did the literature provide some explanations on how and why such impacts were observed? Did the findings reflect some or all of the quadruple aims of healthcare delivery^11^?

In this scoping review, a CBA study is defined as one that involves identifying, measuring and comparing the benefits and costs of an EMR implementation program or project. The costs and benefits need to be presented quantitatively (e.g., in dollars), based on financial and/or economic data, and summarized using net present values (NPVs) or internal rates of returns (IRRs) or returns on investment (ROIs). While studies calculating net financial cost (cost minus revenue) or incremental cost-effectiveness ratio (ICER) were not excluded from the review, they were not classified as CBAs. Additionally, the analyses should reflect an assessment of opportunity costs. That is, any EMR implementation inevitably presented a trade-off of resource uses: diverting scarce resources of labor, capital, and materials from alternative projects (e.g., other interventions or productions of healthcare services) to the EMR project that contributes to increasing volumes and/or quality of healthcare services in the future.

### Stage 2—inclusion criteria and search strategy

The literature search was conducted in February 2021, following the Preferred Reporting Items for Systematic Review and Meta-Analyses (PRISMA) guidelines^12^, supplemented by the PRISMA checklist^13^ for scoping review (see Supplementary Table 2). Search terms are also listed in Supplementary Table 1.

The two main electronic databases used were MEDLINE and Econlit. Additionally, reference lists of identified publications, some key journals (e.g., the Journal of Benefit Cost Analysis) and relevant websites (organizations, conferences, and networks) were hand-searched for additional articles that might meet the inclusion criteria.

The inclusion criteria strictly followed the research questions identified in Stage 1. That is: studies that (i) evaluated EMR or EHR or eHealth implementation for hospitals, (ii) included both benefits and costs; qualitative discussions accepted as we anticipated that many studies could not quantify all the benefits or costs items deemed important, but (iii) reported at least some quantitative results, either costs or benefits; that is, studies discussing benefits or costs without reporting any quantitative values would be excluded.

### Stage 3—study selection

All studies included from the literature search in Stage 2 were imported into Covidence, a platform to conduct literature review in teams (https://www.covidence.org/). The identification of included studies was undertaken by five independent reviewers. First, all duplicates were removed. Second, the first two reviewers (TC, LW) screened the titles and abstracts for eligibility. A study was included if both reviewers agreed that it met the inclusion criteria. Conflicts in reviewers’ conclusions were resolved by discussion and/or examining the full article. Third, full texts of all included studies were reviewed independently by two other reviewers (CW and DS). Reference lists of the included studies were screened by two reviewers (CW and DS) to identify further studies that might have been missed. Published systematic reviews and meta-analyses were examined to ensure that no eligible studies were missed. Both reviewers systematically and independently documented the properties of the included studies and developed their own data extraction tables and notes. Data extraction fields were then compared, discussed and finalized. Each reviewer completed the data extraction process independently, following the agreed data extraction format.

From steps 2 to 4, the screening quality was controlled by an independent reviewer (KHN), who randomly selected and screened papers independently for inclusion/exclusion. All reviewers of each round met, compared and discussed their independent decisions to reach final agreement.

### Stage 4—data extraction from included studies

We documented characteristics and quantitative information in each study to synthesize findings. Quantitative information was sorted by key analytical features and themes. All data were entered onto a “data charting form” that recorded both general information about the study (author, year, country, and setting) and specific information related to CBA of interest, that is, analytical method and duration, benefit and cost items, analysis perspective, data type, sample size (if any), statistical methods used (if any), and quantitative main findings.

### Stage 5—collation of data and findings

Using the charted data, we grouped the similar findings into common themes to answer the research questions formulated in Stage 1. All benefit and cost items documented in this literature were described and then grouped into categories. We did not limit the number of categories or the number of items to be included in each category in order to understand the diversity in benefit and cost definitions and measures. We reported precisely what were used, from the information available in the studies (including their appendices, if any), and allowed for the themes to emerge naturally.

While interested in benefit and cost values, we did not attempt to conduct meta-regression analysis or “quantitatively synthesize” those values. The quantitative results were, however, summarized and recorded for each study. This is consistent with the scoping review approach whereby quantitative and quality assessment was not the main objective. However, we provided some judgements on whether or not the literature provided robust or generalizable findings that can be useful for future CBA of EMR implementation in hospital settings.

Finally, we attempted to map the objectives and evaluation findings of all studies to the quadruple aims of healthcare delivery. That is, improve population health, enhance patient experience, reduce cost per patient, and improve work-life balance of the healthcare workforce^11^. Since EMR, like any other healthcare infrastructure and investment, has been developed to achieve the ultimate outcomes of healthcare delivery, it is crucial to benchmark the EMR implementation against these aims.

## Results

### Summary of included studies

**Fig. 2:**
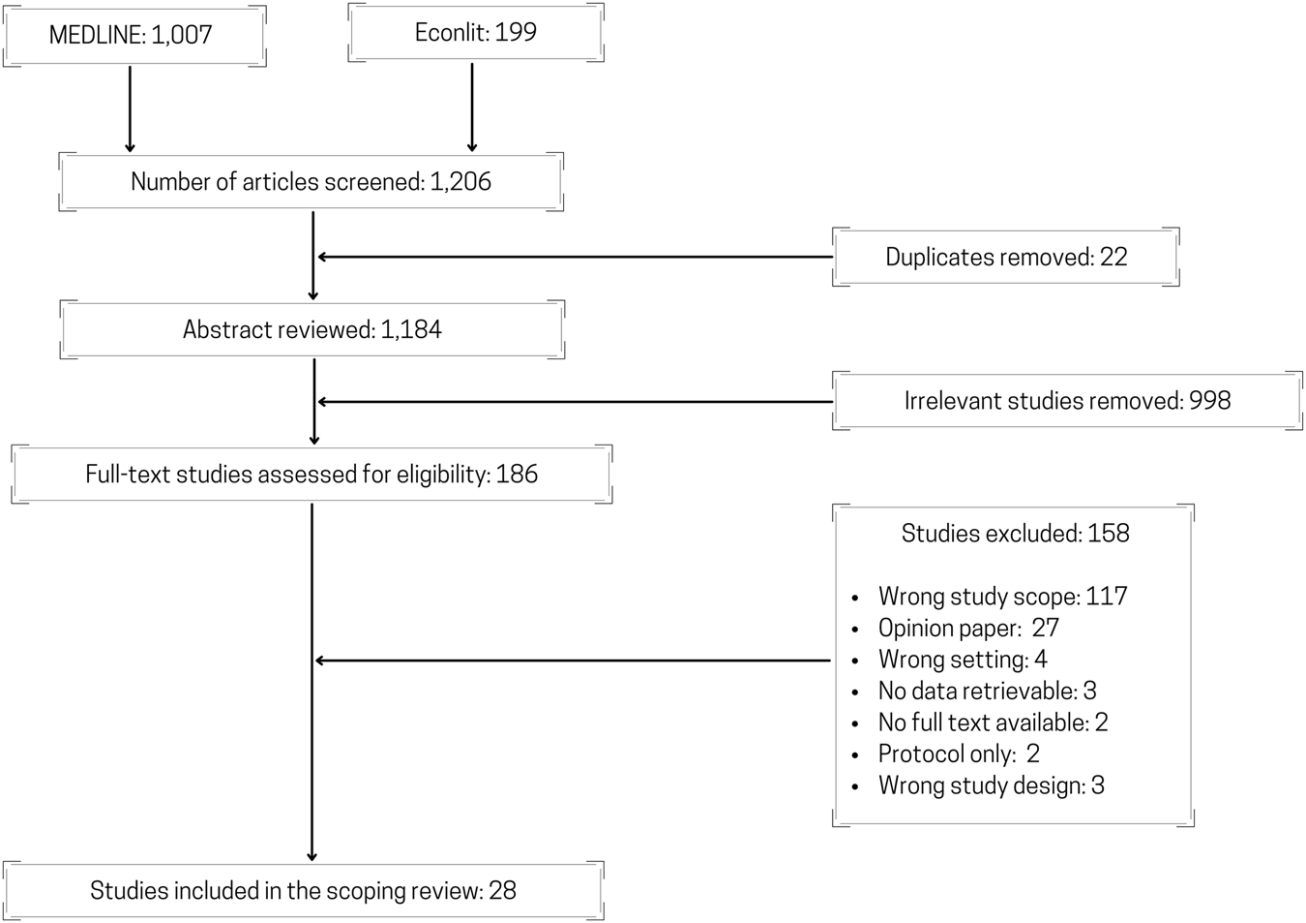
The Preferred Reporting Items for Systematic reviews and Meta-Analyses extension for Scoping Reviews (PRISMA-ScR) flowchart.

The scoping review search resulted in 1184 unique articles. Through the abstract and full-text screening process, a final list of 28 articles were collated. A summary of all 28 included articles is included in Table 1, and the reviewed process is summarized in the PRISMA diagram (Fig. 2). All studies were numbered (see the numbering in Supplementary References) and referred to in the following sections.

**Table 1:** Characteristics and overview of included studies.

ID	Authors	Study type	Type of EMR systems	Country of study	Data year	Research questions	Methods of analysis / evaluation
3	Beresniak et al. (2016)	CBA	EHR4CR	Europe/Global	2012–2013	To conduct a CBA to assess the potential added value of EHR4CR in the pharmaceutical industry compared to current practice, utilizing oncology clinical trials as a reference case	Cost benefit analysis
2	Li et al. (2012)	CBA	EMR	China	2006–2010	To conduct a perspective cost-benefit study to analyze the financial effects of EMR implementation compared to traditional paper records	Cost benefit analysis
1	Choi et al. (2013)	CBA	EMR	Korea	2006–2012	To analyze the economic effects of EMR in a hospital by conducting a CBA on differential costs of managerial accounting	Cost benefit analysis
11	Nuckols et al. (2015)	Cost effectiveness analysis	CPOE	USA	2013	To determine the probability that a CPOE system creates societal financial savings upon implementation	Probabilistic model
12	Sevick et al. (2017)	Cost effectiveness analysis	EDCT	Canada	2012–2013	To complete an economic evaluation within a randomized controlled trial comparing the use of an electronic discharge communication tool compared with usual care.	Bootstrapping analysis and sensitivity analysis
15	Ben-Assuli et al. (2016)	Cost effectiveness analysis	EHR	Israel	2016	To investigate the relationship between EHR and the financial and clinical outcomes in an emergency department through the use of simulation and Markov model	Markov model
13	Spaulding et al. (2013)	Cost study	CPOE	USA	2007	To assess the impact of CPOE systems usage on cost and process quality in the medication management process	Heckman selection correction to create ordered Probit model
10	Kazley et al. (2014)	Cost Study	EHR	USA	2009	To determine whether advanced EHR use in hospitals is associated with lower cost of providing inpatient care.	Generalized linear model
14	Teufel et al. (2012)	Cost study	EMR	USA	2009	To determine whether delivering care with advanced-stage EMR was associated with a decreased cost per case in a national sample of hospitalized children	Bivariate analysis
6	Dranove et al. (2014)	Cost study	EMR	USA	1996–2009	To conduct an empirical examination of the impact of EMR adoption on hospital operating cos	Linear regression with fixed effects
5	Atasoy et al. (2018)	Cost study	EHR	USA	1998–2010	To analyze the spill over effects of EHR adoption of each hospital on the costs of neighboring hospitals in the same Health Service Area (HSA) and identify if spill over effects occur	Econometric modeling
27	Zhivan et al. (2012)	Efficiency and productivity analysis	CPOE	USA	2006	To empirically examine the association between hospital inefficiency and the decision to introduce EMRs and CPOEs in a national sample of U.S. general hospitals in urban areas in 2006	Logistic regression
18	Eastaugh et al. (2012)	Efficiency and productivity analysis	EHR	USA	2008–2011	To determine what factors, increase the adoption rate of EHR in hospitals and to assess the effectiveness of the system in staff scheduling	Logit regression
9	Furukawa et al. (2010)	Efficiency and productivity analysis	EMR	USA	1998–2007	To examine the impact of EHRs on cost efficiency in hospital medical-surgical units	Stochastic Frontier analysis
8	Dupont et al. (2017)	Financial analysis	EHR4CR	Europe	2007–201	To assess the financial sustainability of exploiting the EHR4CR platform from the perspective of an EHR4CR service provider in the context of clinical research	Market analysis, value change, business model simulation
7	Driessen et al. (2013)	Financial analysis	EMR	Malawi	2010–2011	To model the financial effects of implementing a hospital wide EMR system in a tertiary facility in Malawi	Financial discounting using actual cost and counter-factual data
17	Gowrisankaran et al. (2016)	Impact Study	CPOE	USA	2006–2010	To evaluate if the adoption of EHRs leads to increases in billing practices	Econometric modeling
22	Haque et al. (2015)	Impact study	CPOE & EMR	USA	2008–2011	To determine the impact of EMRs on the clinical outcomes of patients	Difference in differences model
24	Hydari et al. (2019)	Impact study	CPOE & EMR	USA	2005–2014	To determine the impact that advanced EMRs have on patient safety events	Difference in differences method
21	Freedman et al. (2014)	Impact Study	CPOE & PD	USA	2003–2010	To study the effect of EMR hospital adoption on patient safety and health indicators	Empirical model created through econometrics techniques
16	DesRoches et al. (2010)	Impact Study	EHR	USA	2008–2009	To assess whether EHR adoption was linked with better performance on standard process-of-care measures including lower mortality, readmissions rates, length of stay and inpatient costs	Multivariate model
4	Iturrate et al. (2016)	Impact Study	EHR with modified ordering system	USA	2013–2015	To observe the change in laboratory utilization after the repeat order system on EHR is disabled	Pre- and post-intervention as a continuous variable with an approximately normal distribution using Student’s *t* test. Additionally, we used an interrupted time series design and performed segmented regression analysis, which divided our time series into pre- and postintervention segments.
28	Zlabek et al. (2011)	Impact Study	EHR, CPOE	USA	2007–2009	To examine the effects of an inpatient EHR system with CPOE on selected measures of cost of care and patient safety	Descriptive statistics with t-tests and chi-squared tests
25	Miller et al. (2011)	Impact Study	EMR	USA	1995–2006	To assess the impact of EMR adoption on neonatal mortality rates	Panel model with fixed effects
26	Xue et al. (2012)	Impact study	EMR	China	2005–2009	To evaluate the impact of the EMR system on efficiency, quality, and cost of inpatient care in the hospital	Interrupted time series analysis
20	Encinosa et al. (2012)	Impact Study	EMR	USA	2001–2007	To assess the impact of EMR on patient safety once a patient safety event has occurred	Multivariate regression analysis
19	Encinosa et al. (2013)	Impact Study	EMR	USA	2010	To examine what impact the adoption of meaningful EMR usage has on hospital-acquired adverse drug events and their costs in 2010	Regression analysis
23	Himmelstein et al. (2010)	Impact study	EHR	USA	2003–2007	To assess if there is a correlation with a hospital’s computerization score and the cost or quality of patient care	Bivariate analysis; Multivariate analysis

*EMR* electronic medical records, *EHR* electronic health records, *EHR4CR* electronic health records for clinical research, *CPOE* computerized physician order entry, *EDCT* electronic discharge communication tool, *PHR* personal health record.

While the investigated EMR systems were varied, the majority (17/28)^(ID#1-2,#6-7,#9,#11-14,#17,#19-22,#24-27)^ were categorized under the EMR definition provided by *Dranove*, et al. (2014)^14^, which includes CPOE. A further eight studies being classified as EHR systems^(ID#4-5,#10,#15,#16,#18,#23,#28)^ and two EHR for clinical research (EHR4CR) ^(ID#3,#8)^ systems were also included within the scope of the literature review.

Studies were all published between 2010 and 2019, with an average of three studies published each year. Generally, there were fewer studies published in more recent years; most studies were published between 2010 and 2014 (17/28). Across the literature, the data used to estimate benefits and costs dated back from 1995 to 2016, with most data from 2000 to 2010. Twenty studies were undertaken in the US^(ID#4-#6,#9-#11,#13-14,#16-25,#27-28)^, with the remaining studies from OECD countries (Canada^(ID#12)^, Korea^(ID#1)^, Israel ^(ID#15)^, and the European Union^(ID#3,#8)^) and three from middle- and lower-income countries (China^(ID#2,#26)^, Malawi^(ID#7)^).

### Types of economic analysis or evaluation (Q1)

Studies were classified into four categories: cost–benefit analyses (CBA), cost studies (including financial analyses), impact evaluation (primarily focusing on hospital or patient outcomes), and effectiveness and efficiency analyses (including cost-effectiveness analyses, cost efficiency, and productivity analyses).

Despite a relatively rich literature on the impact of EMR implementation, only three studies^(ID#1-#3)^ were qualified as (full) CBA studies (following the definition in Stage 1). The unit of analysis is a hospital where EMR was implemented. These studies presented benefits and costs in dollar values and presented estimates of net benefit. Some benefit and cost items were valued using nonmarket prices (i.e., economic or shadow prices) as opposed to financial values (or market prices). A short description of these studies is presented in Box 1.

The second category—“cost studies”, including financial analyses—primarily examined the cost or financial expenses of EMR systems (7/28)^(ID#5-8,#10,#13-14)^. Among those, some had a slight feature of a CBA by incorporating both aspects cost and revenue resulting from EMR implementation. The reported results were often in net cost or differences in cost between EMR-based hospitals and those without or pre EMR. These studies were conducted at both patient and hospital level, i.e., the unit of analysis was either hospitals or individual patients admitted to hospitals.

“Impact evaluation” studies examining the outcomes by stakeholders (either outcomes for patients, or staff, hospitals, society, or health system) resulting from the EMR implementation: 12 studies out of 28 ^(ID#4,#16-17,#19-26,#28)^. Studies looking at “cost-savings” were included in this group when the cost-savings were described as a result of changed/improved (patient) outcomes or reduced wastes. Similar to cost studies, the unit of analysis was either individual patients or hospitals.

Last, we grouped “cost effectiveness” and “efficiency and productivity analysis” studies into the last category (6/28 studies). These studies considered both the cost and outcomes of EMR implementations (i.e., full evaluation) but did not quantify them in terms of monetary values (hence not CBA). Three cost-effectiveness analyses^(ID#11–12,#15)^ estimated costs, quality-adjusted life years (QALYs), and presented incremental cost-effectiveness ratio (ICERs) for a targeted hospital patient population. Two studies^(ID#9,#27)^ examined cost efficiency resulting from EMR implementation and one investigated labor (nurse) productivity improvement^(ID#18)^. The unit of analysis in these studies was hospitals in the United States.

Key information for all studies is presented in Tables 1 and 2, and findings of the three CBAs are summarized in Box 1.

**Table 2:** PICO characteristics of included studies.

ID	Authors	P (population)	I (intervention)	C (comparator)	O (outcomes)	Quantitative findings	Outlook on EMR
3	Beresniak et al. (2016)	Oncology sector	EHR4CR	Current practice	Expected net benefit	The expected benefits were estimated at €161.5 m (clinical scenario S1, protocol feasibility assessment), €45.7 m (clinical scenario S2, patient identification for recruitment), €204.5 m (S1 + S2), €1906 m (clinical scenario S3, clinical study execution), and up to €2121.8 m (S1 + S2 + S3)	Positive
2	Li et al. (2012)	Hospital patients	EMR	Paper-based medical record	NPV, ROI	The net benefit (total) from EMR implementation for a 6-year period was $559,025 in the general hospital. The time of return on investment is 3 years; and the pessimistic time of return on investment is 5.38 years.	Positive
1	Choi et al. (2013)	Outpatients	EMR	Paper-based medical record	NPV, BCR, DPP	The estimated NPV was US$3,617,000 for an 8-year period. The estimated BCR was 1.23. The estimated DPP was about 6.18 years.	Positive
11	Nuckols et al. (2015)	Inpatients	CPOE	Paper ordering system	Cost, QALY, ICER	CPOE, on average, had 99% probability of yielding savings to society and improving health, compared to the paper ordering system. Per hospital (by size, approximated by number of beds), mean lifetime savings –in millions- were $11.6 (25-72 beds), $34.4 (72-141 beds), $71.8 (141-267 beds), and $170 (267-2,249 beds) (2012 dollars). Quality-adjusted life-years (QALYs) gained were 19.9, 53.7, 109, and 249, respectively. Nationwide, anticipated increases in CPOE implementation from 2009 through 2015 could save $133 billion and 201,000 QALYs.	Positive
12	Sevick et al. (2017)	Inpatients	EDCT	Usual care	Cost, QALY, ICER	The incremental cost effectiveness ratio (cost per QALY gained) was estimated at $C239,933 for EDCT arm compared with usual care. There was a small gain in effectiveness and approximately $C800 difference in resource utilization costs.	Positive
15	Ben-Assuli et al. (2016)	Patients in ED	EHR	Without EHR	Cost, QALY, ICER	The incremental cost effectiveness ratio (cost per QALY gained) was estimated at $1,228.52 when the EHR system was made available to physicians compared to when EHR was not available.	Positive
13	Spaulding et al. (2013)	Hospitals	CPOE	Before CPOE	Cost	Even when 100% CPOE usage was not attained in hospitals, there remain benefits. From 51- 90% usage is associated with the lowest predicted nursing cost costs per patient day. A large increase in nursing salaries was associated with 91-100% usage and the most beneficial cost outcomes accrue at under 50% usage for the pharmacy.	Positive
10	Kazley et al. (2014)	Inpatients	EHR	Without EHR	Cost	After accounting for variations in patient and hospital characteristics, it was estimated that on average patients treated in hospitals with advanced EHRs cost $731 (approximately 9.66%) less than patients admitted to hospitals without advanced EHRs.	Positive
14	Teufel et al. (2012)	Pediatric patients	EMR	Without EMR	Cost	EMR is creating a safer health care system but not always associated with inpatient cost savings. Advanced stage EMR was associated with an average 7% greater cost per case ($146 per discharge).	Neutral
6	Dranove et al. (2014)	Hospitals	EMR	Without EMR	Operating cost	EMR adoption is initially associated with a rise in cost. EMR adoption at hospitals in IT-intensive locations leads to a decrease in costs after 3 years. Hospitals in other locations experience an increase in costs even after 6 years.	Neutral
5	Atasoy et al. (2018)	Hospitals	EHR	Without EHR	Operating cost	The adoption of an additional EHR system in the focal hospital increases its own costs 1.8% in the current year and 2.3% in 4 years. If hospitals in the same HSA (neighboring hospitals). The adoption an additional HER system corresponds to 1% decrease in the costs of the focal hospital in the current year, and a cumulative effect of 1.5% decrease in four years.	Positive
27	Zhivan et al. (2012)	Hospitals	CPOE	Without CPOE	Cost	Hospitals, on average, exceeded costs at the frontier by 16%. The hospital cost-inefficiency is positively related to the EMR adoption decision, but not CPOE adoption. An 1%-point increase in inefficiency score was associated with a 3.3% increase in the odds of EMR adoption.	Neutral
18	Eastaugh et al. (2012)	Hospitals	EHR	Without EHR	Nursing productivity	It was estimated that 25% improvement in financial health of a hospital is associated with 5.1% increase in EHR. A 25% increase in school dependency on the hospital as a source of clinical rotation leads to a 2.0% increase in EHR. The implementation of EHR was associated with a 1.6% improvement in productivity.	Positive
9	Furukawa et al. (2010)	Surgical units in hospital	EMR	Without EMR	Cost	EMR stages 1 and 2 were associated with significantly higher inefficiency scores. EMR stage 3 shows no or negative association with inefficiency, depending on the estimation models used. It was concluded that EMR, overall, is associated with higher inefficiency in medical-surgical acute settings	Negative
8	Dupont et al. (2017)	Service providers	EHR4CR	Without EHR4CR	Cost, Revenue	It was estimated that a profitability ratio 1.8 or higher could be achieved at year 1. There are potential for growth for the ratio in subsequent years if the market uptake is higher.	Positive
7	Driessen et al. (2013)	Inpatients	EMR	Without EMR	Cost	It was estimated that the total cost savings US$284,395 annually (for in length of stay, transcription time, and laboratory use). There is a net financial gain by year 3, after accounting for the costs of installing and sustaining the EMR system. The estimated cost savings was US$613,681 over the 5 years.	Positive
17	Gowrisankaran et al. (2016)	Hospitals	EMR (CPOE)	Without EMR (CPOE)	Administrative practice in hospital (upcoding of medical and surgical procedures)	EMR adoption in hospital led to increases in reported severity for medical relative to surgical patients at EMR hospitals because EMRs decreases coding costs for medical patients. Medicare costs might increase by $689.6 million annually with post-reform completeness of coding with EMRs. There was a positive and significant impact from EMR adoption on the mean DRG weight following the reform.	Neutral
22	Haque et al. (2015)	Patients	EMR	Without EMR	Length of stay, thirty-day mortality, thirty-day readmission	It was found that EMRs had the largest impact for relatively less-complex patients. Admission to a hospital with an EMR is associated with a 2% reduction in length of stay and a 9% reduction in thirty-day mortality for less complex patients. In contrast, there was no evidence of statistically significant benefit for more-complex patients in hospital with EMR.	Positive
24	Hydari et al. (2019)	Patients	CPOE & EMR	Without CPOE & EMR	Patient safety events (medication errors, falls, complications)	EMRs were found to lead to a 17.5% decline in patient safety events, driven by reductions in medication errors, falls, and complication errors. There was also a decline in medium- and high-severity events with advanced EMRs.	Positive
21	Freedman et al. (2014)	Inpatients	EMR (CPOE & PD)	Without EMR (CPOE & PD)	Preventable adverse events	There was evidence that EMRs improve patient safety (reduced the likelihood of adverse events), particularly for less complex patients. Adoption of CPOE was associated with an 11% drop in the probability of experiencing at least one postoperative adverse event for cases with no more than one comorbidity and a 17% drop in probability for patients with more common DRGs. The results indicated EMR is likely to have the greatest impact on patient safety indicators when the technology has a decision support feature that is relevant and accurate for the patient’s condition.	Positive
16	DesRoches et al. (2010)	Hospitals	EHR	Without EHR	Quality of care, risk-adjusted length of stay, readmission rate, cost	The relationship between quality and efficiency were modest at best and not statistically significant. Hospitals with EHR had slightly better performance on prevention of surgical complication (93.7% for hospitals with comprehensive EHR, compared to 93.3% with basic EHR, and 92% without). Length of stay was about 0.5 days shorter for cases of pneumonia in hospitals with comprehensive EHR compared to those without. Inpatient costs are comparable across hospitals with and without EHR.	Neutral
4	Iturrate et al. (2016)	Patients requiring lab tests	EHR with modified ordering system	EHR without modification	Lab test per patient adjusted for outcome	Following introduction of the modified EHR ordering system there was a significant reduction in target lab tests per patient day. Segmented regression analysis indicated a 20.9% reduction in the utilization of target lab tests. Student’s t test analysis indicated a .5% reduction. The estimated reduction in hospital costs was $300,000 due to the EHR modification.	Positive
28	Zlabek et al. (2011)	Service records in hospital	EHR, CPOE	Without EHR and CPOE	Lab test, radiology examinations, paper consumption, transcripts, medication errors and near misses,	Laboratory tests per week per hospitalization decreased about 18% (from 13.9 to 11.4). Radiology examinations per hospitalization decreased 6.3% (from 2.06 to 1.93). Monthly transcription costs declined 74.6% (from $74 596 to $18 938). Reams of copy paper ordered per month decreased 26.6% (from 1668 to 1224). Medication errors per 1000 hospital days decreased 14.0% (from 17.9 to 15.4). Near misses per 1000 hospital days increased 38.9% (from 9.0 to 12.5). The percentage of medication events that were medication errors decreased from 66.5% to 55.2%.	Positive
25	Miller et al. (2011)	Babies	EMR	Without EMR	Neonatal mortality	EMRs increase speed and accuracy of access to patient records, leading to improved diagnosis and monitoring. It was estimated that a 10% increase in births that occur in hospitals with EMR reduces neonatal mortality by 16 deaths per 100,000 live births. The estimated cost-effectiveness suggested that EMR was associated with $531,000 per baby’s life saved.	Positive
26	Xue et al. (2012)	Inpatients	EMR	Without EMR	Length of stay, infection rate, mortality rate, and cost per inpatient	EMR was associated with reduced length of stay, infection rate, and mortality rate but had no correlation with patient costs. Length of stay grew at 0.027 bed-days per month in the pre-EMR period and declined at 0.043 bed-days per month in the post-EMR period. Infection rate rose at 0.036 infections per 100 patients per month in the pre-EMR period and declined at 0.062 infections per 100 patients per month in the post-EMR period. Mortality rate grew at 0.048 deaths per 1000 patients per month in the pre-EMR period and decreased at 0.005 deaths per 1000 patients per month in the post-EMR period. Cost per patient stay declined at 33 RMB per month in the pre-EMR period and increased at 16 RMB per month in the post-EMR period.	Neutral
20	Encinosa et al. (2012)	Adverse events in patients	EMR	Without EMR	Death, 90-day readmission for surgeries, 90-day hospital expenditure for surgeries	While EMRs did not reduce the rate of patient safety events, they reduce death by 34%, readmissions by 39%, and spending by $4,850 (16%) if a safety event occurred. This led to a cost offset of $1.75 per $1 spent on IT capital.	Positive
19	Encinosa et al. (2013)	Inpatients	EMR with meaningful use (MU) requirements	EMR without MU	Hospital acquired adverse events	It was estimated that hospital cost savings per averted adverse events were $4,790. If all hospitals in Florida had adopted all 5 functions in the EMR, 55,700 ADEs would have been averted and $267 million per year would have been saved. The cost savings was estimated to recoup only 22% of information technology costs.	Neutral
23	Himmelstein et al. (2010)	Hospitals	EHR	Without EHR	Quality of care, administrative costs as share of total cost	Hospitals on the “Most Wired” list performed equally compared to others on quality, costs, or administrative costs. Hospital computing however might modestly improve process measures of quality.	Neutral

*ED* emergency department, *EMR* electronic medical records, *EHR* electronic health records, *EHR4CR* electronic health records for clinical research, *CPOE* computerized physician order entry, *PD* physician documentation, *EDCT* electronic discharge communication tool, *PHR* personal health record, *NPV* net present value, *BCR* benefit cost ratio, *DPP* discounted payback period, *ROI* return on investment, *QALY* quality adjusted life year, *ICER* incremental cost-effectiveness ratio.

Box 1 Summary of the three full CBAs in the EMR evaluation literature **ID#1:** Choi et al. (2013), performed a CBA following the implementation of an outpatient EMR system at the Samsung Medical Center in Korea in 2008. The study focused on the financial costs and benefits separated into 4 categories: system costs for implementation of the EMR system, induced ongoing costs to assist in the transition to an EMR database, cost reductions from resource savings, and additional revenues from the opportunity cost savings. Overall, the largest financial benefit was incurred from increasing outpatient capacity. This contributed to a positive NPV after 6 years of operation, indicating financial viability of EMR systems, even when disregarding the potential for the systems to improve patient care.

**ID#2:** Li et al. (2012), similarly presented another CBA to assess the financial viability of an EMR system in a Chinese hospital in 2010. Like Choi (2013), the main emphasis was on the financial benefits; however, this study also considered the financial cost savings from reduction in adverse drug events and medication errors. Although nonfinancial benefits were not considered, a positive net benefit was calculated after 3 years of EMR operation.

**ID#3:** Compared with the previous two studies, Beresniak et al. (2016), only addressed the costs and benefits that EMR data would have in clinical research for the global pharmaceutical oncology sector. Overall, 18 benefits were identified, occurring across the initial feasibility stage of clinical studies, patient identification stage in clinical studies, and the execution stage of clinical studies. The key benefit identified within this study was that a comprehensive EMR system would allow the pharmaceutical industry to have a quicker time to market for newly developed drugs, with a large NPV associated with the EMR system.

### Evaluation scope and measurements of benefits and costs

As shown in Table 2, the unit of analyses varied from services (lab tests, examinations) to patients, hospitals, and hospital sector. The variation naturally led to different choices of measurements and analytical methods. Indicators that measure the same impacts (e.g., clinical outcomes such as adverse events or mortality), were defined at both patient level (disaggregate) and organization/system level (aggregate).

Impacts could be sorted into six domains, which were then mapped to the quadruple aims of health care (see Table 3, and further discussion on the quadruple aims in the “Discussion” section): (1) patient outcomes and experience, (2) hospital outcomes, (3) health-system (sector) outcomes, (4) cost specific to EMR implementation, (5) hospital resource utilization and (6) productivity and efficiency measures. These six domains have some overlaps because many indicators can be used to measure outcomes at multiple levels: patient, organization/hospital, and health system/sector. As shown in Table 3, within each domain, multiple measures and instruments were used for the key outcomes of interest. For instance, death/mortality risk and adverse events are key outcomes for patients, hospitals, and population health. Specifically, within “patient outcomes” domain, the impact of EMR on adverse drug events (ADEs) was measured by the counts of ADEs in elderly patients or less complex patients; when examined at the hospital level (i.e., hospital outcomes domain), the impact of EMR on patient safety was then measured by the mortality rate after ADEs. Similarly, cost per episode of care can be measured by admission (patient level) or as average cost per patient (hospital level), or cost per casemix-adjusted episode of care (health-system level). The partial productivity measures (efficiency and productivity index) are special cases where both outcomes and costs were included in the measure, therefore, warranting its own domain.

**Table 3:** Impact domains and alignment of studies to quadruple health care aim (Bodenheimer and Sinsky, 2014)^11^.

Key impact domains	Measures and/or indicators	Mapping to the quadruple aims of health care delivery
Patient outcomes and experience	Neonatal deaths; prematurity deaths; Counts of patient safety events; Adverse drug events for: elderly, more common, less complex patients; Count or rate of medication errors; near-missed events avoided; Iatrogenic injury avoidance. Patient pain score, patient anxiety score; patient confidence in healthcare system rating; Quality adjusted life years;	- Improve population health - Enhance patient experience
Hospital outcomes	Change in mortality rate; Mortality rate after adverse drug event; 30-day mortality rate; Rate of readmission; reduced readmission rates; changes of infection rate; Length of stay for repeat patients; Length of stay (rate of change for); Length of stay for less complex patients. Quality of care provided; Acute myocardial quality scores; Pneumonia quality score; Congestive heart failure quality score; Composite quality score.	- Improve population health - Enhance patient experience - Reduce cost per patient
Health system outcomes	Crude mortality per 1000 population for: hypertension related mortality; maternal related mortality; infant related mortality; child 1-5 mortality; child 1-5 HIV deaths attributable to mother to child transmission of HIV; adverse drug related mortality; acute respiratory infection related mortality in over five years.	- Improve population health
Cost specific for EMR implementation	Initial capital costs for: Development; Implementation; Training; Hardware; Marketing; Electronics; Office supplies. Ongoing costs for: Paper scanning system; New medical record creation; information technology support; Medical transcriptionists; Maintenance; Software; Meeting times;	- Reduce cost per patient
Hospital resource utilization	Laboratory tests per week; Radiology examinations; Iatrogenic testing; Head CT scans; Chest radiographs; Body CT scans; Consultations; Prescriptions; Paper storage space reutilization; Litigation cost; Medicare spending; Labor costs; Redundant employees; Nursing and pharmacy salary costs; Nursing scheduling; Physician workload; Administration time	- Reduce cost per patient
Productivity and efficiency measures	Inefficiency scores; productivity change over time; Rate of change in cost per patient; Per patient costs; Outpatient average spending;	- Reduce cost per patient

Nonetheless, there are some clear demarcations between these domains. Patient outcomes were primarily observed through deaths (count of), adverse events (count of, or rate of), quality adjusted life years (QALYs), and outcomes at the organization level (hospital) focused on key performance indicators such as “length of stay” and “medication errors” (count of, or rate of) or “inefficiency score”. System-level outcomes (healthcare sector and society, overall) have been on population-based “mortality rates” (of mother, children, and older people). A large number of studies examined the costs of EMR implementation through initial upfront monetary costs (financial investment) and operating (recurrent) expenditure. These are mostly financial analyses of EMR impact. The domain of “hospital resource utilization”, which was associated with cost savings in a large number of studies, focused entirely on changes in tests, procedures, and consumable goods (including paper, medical supplies, and prescriptions). These studies might quantify the impact into cost, but the primary interested was the estimation of resource savings. However, it is noted that very few studies provide unambiguous definitions of the indicators used to measure impacts.

### Findings on the net benefit or net cost (Q3)

The studies generally indicate that there is a positive overall impact from the EMR implementation (see summarized quantitative findings and outlook on EMR, Table 2). Of the 28 studies, 19 indicated positive outcomes from the implementation of EMR and 8 indicated mixed outcomes from EMR implementation, often citing a trade-off between increased quality of care and increasing costs. Furukawa, et al. (2010) posed the only study that indicated EMR implementation was not cost-effective. This study did not discuss any changes in the quality of care provided.

Most studies that focused on the domains relating to the costs of EMR implementation and resource savings indicated a “lag effect”: it often took approximately 2–3 years to clearly observe the benefits of EMR implementation^(ID#26)^. In addition, three studies indicated that the level of EMR benefits is reliant on the creation of a network effect, both within a hospital ^(ID#13)^ and across a region of hospitals ^(ID#5,#6)^. Notably, the benefits of EMR reported in this literature varied with the date of publication. During the period of 2010–2014, 10 out of 18 studies (56%) indicated that EMR systems might be beneficial for healthcare delivery. However, during the period of 2015–2019, 9 out of 10 studies (90%) were favorable toward the EMR implementation.

## Discussion

In this paper, we have reported findings from the first comprehensive scoping review examining the economic impacts of EMR implementation in the hospital setting using CBAs. We wanted to first understand the types of CBAs used to evaluate EMR implementation; second, to determine how comprehensive the analyses were, and how the benefit and cost items were identified, measured and valued; and third, to determine whether the literature found that the benefits of EMR implementation justify the investment costs.

The review showed that despite a relatively rich ten-year literature, only three studies^(ID#1–#3)^ qualified as CBA studies, the rest focused on impact evaluation, cost/financial analysis, benchmarking (e.g., efficiency analysis) or evaluation of interventions that were EMR-powered^(ID#13–14,#17)^. More than two-thirds of the studies originated from the United States, following the Obama Care boost, and in favor of statistical analysis approaches rather than CBA. Only three studies originated in Europe, while none was from the United Kingdom. Of the three studies conducted in Europe, one of them was a CBA, despite its narrowed scope (impacts of hospital EMR on research and development). The other two CBA studies, in China and Korea, investigated a wider scope of impacts; however, the measurement and valuation of impacts were limited to financial impacts or cost savings from reduced adverse events. Patient outcomes and experience, changes in healthcare workforce as the results of EMR implementation were either not measured or valued. These limitations were acknowledged in all studies.

While most studies pointed toward a positive impact of EMR implementation, such impact is not quantitatively comparable across studies (as summarized and described in Tables 2 and 3). This is attributable to three main reasons: heterogeneity of impact measures (both benefits and costs), different implementation conditions (despite all in the hospital setting), and a large variety of analytical methods employed to understand the economic value of EMR, thus biases associated with those methods. As EMR adoption continues to spread, the reproducibility of findings that allow comparative analyses across settings and healthcare systems is critical to know what works and what does not, how, and why.

First, there were a wide range of analysis units, measures and indicators used to report on the impacts of EMR implementation, from patient to health system levels (as summarized in Tables 2 and 3). Comparable analyses will require increasing standardization of measures of impacts at all levels. Standardization of measures is particularly important as uncertainty existed within the literature as to what mechanisms EMR acts on to create better safety outcomes for patients. *Encinosa et al*. (2012), gave an analogy of EMR acting in the role of a “car airbag” that mitigates damage once an adverse event has occurred, and the CPOE systems act similarly to the role of a car’s “electronic stability controls” in reducing complications^(ID#10)^. While there is substantial evidence that an effective EMR system allows for reductions of medical errors and waste, the causal links from EMR implementation to ultimate patient outcomes such as reduced mortality rate and shorter length of stay are less well understood in the literature. It is proposed that causal links might be better understood if studies can use direct and more precise health outcomes such as vital signs (e.g., body temperature, pulse, and respiration rates) to mediate the impact of EMR on ultimate outcomes (e.g., mortality rates). In any evaluation (especially economic evaluation), understanding the direction and magnitude of change (quantitative) can be equally important as the mechanism of change (e.g., qualitative through understanding the theory of change). The quantitative measures, when measured with standardized instruments across time and implementation units, can highlight the success/failure for lessons learnt and/or further investigations. Additionally, due to current misalignment between evaluation focuses and healthcare aims, the impacts of EMR on quality of care, patient experience, and the healthcare workforce were largely omitted (see Table 3, and further discussion below).

Second, the review identified a very large degree of heterogeneity of implementation conditions and types of EMR systems within the hospital setting. Variance in the latter is especially prominent in studies using large-scale, hospital and panel data. Due to the rapid development of EMR, later adopters were likely to use more sophisticated technologies compared with early adopters, allowing for better capture of the impacts. Variance in the former (across settings/adoption situations) was also noted in the literature, mostly related to the geographical area of adoption. For instance, *Dranove* et al. (2014), demonstrated that the benefits accrued to EMR were reliant on the access to and use of ICT within the local community^(ID#6)^. On the other hand, developing nations and low-efficiency hospitals were shown to benefit from EMR implementation, despite their relatively low level of ICT (see further discussion in theme 6). These seemingly contradicting findings suggest that the large heterogeneity in impact measurement makes it challenging to compare all the results across all the study settings.

Third, studies employed a wide range of economic and statistical methods to understand the value of EMR implementation. These methods ranged from economic evaluation methods such as CBAs^(ID#1–3)^, cost-effectiveness analyses^(ID#11–12,#15)^, and efficiency and productivity analyses^(ID#9,#18,#27)^ to econometric models such as difference-in-difference^(ID#24)^, a system of cost equations^(ID#5)^, or statistical analysis such as multivariate regressions^(ID#6,#11,#12,#13,#16,#19,#20,#21)^, interrupted time series^(ID#4,#26)^, generalized linear models^(ID#10)^, and logistic regressions^(ID#18)^. These models included different sets of variables and measures, many of which were specific to the datasets used or the data-collection context. All studies acknowledged methodology limitations and the potential biases (on the impact estimates of EMR implementation) resulting from the modeling and data limitations. The main sources of biases include self-selection bias of hospitals implementing EMR, self-reporting of EMR usage, and “conservative” estimates of benefits.

Self-selection bias was often examined in studies that utilized longitudinal data, in which investigators attempted to control for the self-selection endogeneity through fixed-effect models. There was a wide range of hypotheses regarding the correlates of EMR adoption in hospitals. Zhivian et al. (2012) proposed that inefficient hospitals were more likely to adopt EMR as the potential improvements were greater than in hospitals that were already more efficient^(ID#27)^. Other studies linked EMR adoption with teaching hospital status^(ID#18)^ or whether the hospital belonged to a community fund-raising scheme^(ID#20)^. As such, it is unclear if the endogeneity would lead to the under- or over-estimation of the EMR impacts, resulting to limited ability to interpolate EMR as a causation for the measured outcomes^(ID#9)^.

EMR use was commonly self-reported both by clinicians and by the organizations collectively (i.e., hospitals)^(ID#16)^. Self-reported savings would inevitably lead to large uncertainty around the estimation of both EMR-related benefits and costs due to self-selection bias. Clinicians who engaged in cost-saving activities could potentially overlook other benefits or costs if they were not directly or clearly related to the cost-saving targets at hand. Conversely, it was believed that hospital response rates were dependent on the successes of EMR, leading to a potential overestimation of EMR benefits.

Many studies stated that they relied on “conservative estimates” when evaluating the benefits of EMR^(ID#1–2,#7,#8)^. Conservative estimates were applied to the loss of productivity following the initial implementation of the EMR system, in two of the CBAs, with predictions for productivity loss higher than expected^(ID#1–2)^. Similarly, studies also used “conservative estimates” in deciding on the counterfactual with costs associated with the counterfactual underestimated to conservatively measure the financial impact from EMR implementation^(ID#7–8)^.

**Fig. 3:**
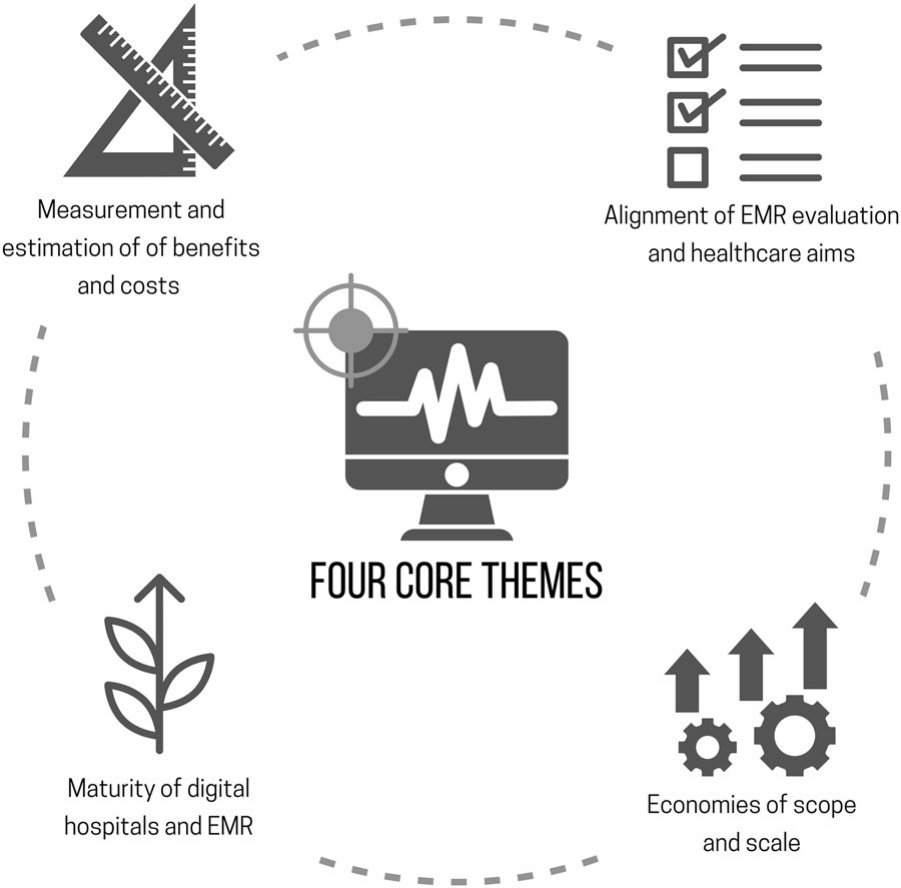
Emerging themes from the literature of economic evaluation and analysis of hospital-based electronic medical records.

Outside the three main questions of the scoping review, we discovered three additional important topics that warrant future research (see Fig. 3). First, the maturity of EMR implementation in the past two decades and how it would affect future evaluation of EMR. Second, the evidence of economies of scale and scope with respect to the impacts of EMR implementation. Third, the (incomplete) alignment of selected evaluation approach/framework with the quadruple aims of healthcare delivery.

### Maturity of EMR implementation

The literature overwhelmingly indicates that most EMR systems were still maturing^15–17^. The full extent of the costs and benefits of EMR implementation could not be understood, measured, and valued using the current metrics or data-collection systems, both were outdated. While publication dates were within the last 11 years (since 2010), most data used for the economic evaluation came from the prior decades (late 1990s–2010).

Despite its relatively short time, a trend has emerged, that is, increasing benefits over time were observed as EMRs slowly matured. Initial studies with data sourced during the late 1990s found that the promised cost-savings from an administration perspective were not delivered through EMR implementation^(ID#6,#9)^. However, later evaluations that accounted for improvement in care quality, using evidence sourced from the 2000s, indicated that EMR provided quality increases at a competitive cost^(ID#14,#25–26)^. In other words, the most recent literature found that EMR is cost-effective. Last, more recent CBAs, sourcing data from the period 2006–2012, indicated that the EMR implementation has become a solution that is financially viable, even when studies did not fully account for improved patient outcomes^(ID#1-3)^.

This change in viability can be attributed to five main factors. First, the capabilities of EMR technology have vastly developed throughout this timeframe. This is perhaps best reflected in the changing role of EMR. Initially, the system was developed and utilized as a system to keep track of resource utilization, costs, and outcomes^(ID#6)^; however, improvements in technology have enabled EMR to provide clinical support and hence provide greater utility to hospitals^(ID#22)^. In parallel, the system has been refined over time to improve the cost-saving utilization. ^(ID#4,#13)^. Second, clinicians have become more accepting of EMR implementation over time. Clinician uptake and technical ability have been marked as a key determinant in the success of EMRs. Third, the role of network effects has led to increasing benefits and decreasing costs associated with EMR^(ID#5)^ Fourth, there is a potential that EMR can contribute to developing improved procedures and workflow through increasing the amount of information available. Furthermore, there is the potential of EMR to be used as the building block for improving medical technologies through the emergence of predictive and prescriptive analytics driven by machine learning and artificial intelligent developments.

In addition to the natural progression of EMR maturity, developments have been made in the systems to support the EMR implementation, which together has led to increasing benefits over time. In the literature, this was often discussed as “workforce training” and “development of ICT services”. Although the perceptions of physicians towards the benefits of EMR remain widely predetermined^18^, larger productivity gains can be made through EMR, especially when physicians are incentivized by sharing in the benefits from the gains^(ID#18)^ However, EMR implementation has been consistently associated with an implementation lag where disbenefits (e.g., lower productivity) are accrued during the training phase^(ID#2,#6)^. The duration of the disbenefits in hospital efficiency studies has been linked with the level of general ICT adoption within a region, suggesting that the prevalence of complementary ICT skills within the community acts to both minimize system costs, and to increase benefits. As such, an increasing use of ICT systems in all operating aspects of society in the future is expected to further benefit the EMR systems.

### Returns to scale and scope

Another common theme relating to returns to scale and scope is that present EMR technologies offer highly effective solutions for simple medical problems but show decreasing effectiveness for highly complex problems. In economic terms, studies found strong evidence of increasing returns to scale and scope of the EMR implementation in small and simple hospital systems but decreasing returns (approaching negative) in large and complex systems. The evidence was visible at both national health system and within the context of a hospital. For instance, great success has been observed in the implementation of the EMR in the Kumuzu Central hospital in Malawi^(ID#7)^. In these systems, with their relatively low stock of ICT capital and labor available, the marginal returns (e.g., improved patient processing and treatment efficiency) from the EMR adoption were significant. Similarly, a US study noted a phenomenon that EMR applications showed their highest effectiveness in the scenario of noncomplex cases^(ID#22)^.

The mechanism driving increasing returns to scale and scope for simple and small medical problems or systems is intuitive. For instance, if all patient information is well captured and linked in a unified EMR system (between departments within a hospital, or across hospitals within a health district/region), when a particular patient repeatedly presents at different locations, it requires less time spent on collecting past medical history and relevant personal information—examples include allergies, comorbidities and complexities. This allows faster and more effective treatment decisions while accruing much lower cost of acquiring patient information (i.e., cost of both medical staff and patient’s time and effort). However, the mechanism that drives decreasing (even negative) returns scale and scope, i.e., EMR showing minimal additional effectiveness in complex cases, is less certain. A common explanation in the literature centers around the lack of precision measures of outcomes in complex situations, such as the contribution of EMR implementation on mortality rate and length of stay^(ID#21–22)^. For example, medication errors that can be effectively reduced by an EMR system might contribute heavily to the mortality risk of patient of simple casemix; however, a patient with a complex casemix might have very high mortality risk, regardless of whether or not medication errors occur.

### The alignment of EMR evaluation with the quadruple aims of healthcare delivery

Our review found that the current literature of economic evaluation of EMR did not sufficiently address the quadruple aim of healthcare delivery, that is, improve population health, enhance patient experience, reduce cost per patient, and improve work-life balance of the healthcare workforce^11^.

Of the 28 studies included in the scoping review, no study explicitly addressed all aspects of the quadruple aim of health care, particularly the impacts of EMR implementation on the healthcare workforce (see Table 3). Considering the high levels of clinician burnout partially attributed to EMR often discussed in noneconomic studies, a gap in the economic literature exists: that is, quantifying the impact of EMR on the work-life balance of the healthcare workforce. Another important aim of healthcare delivery—patient experience—was addressed in only one study. Hydari et al. (2019)^(ID#24)^ observed qualitative benefits in “increased confidence in the health system” (by patients) after EMR adoption, no studies contemplated the patient experience beyond health outcomes or quality-adjusted life years (through the QALY estimates).

A potential explanation for this relatively narrow focus of EMR evaluation to date is the maturity of digital health across settings and countries. The economic development and project-management literatures have shown that piece-meal project implementations, even with best intentions in mind, but without proper alignment to overall goals and purposes of the sector, can result in high wastage and undesirable impacts, especially when projects compete for the same labor and capital resources to produce the same outputs^19,20^.

Project implementations face constraints of time, budget (allocated funding and inputs), and set target outputs/results to achieve (number of staff trained, number of machines installed, or go live dates, etc.). If their success is defined by a narrow set of input–output metrics, rather than measures aligned with sector purposes (i.e., who will benefit, what will change systematically as a result of the project), and goals (i.e., broader sector goals of healthier population, improved patient and staff experience, and reduced cost per capita), many projects might achieve their targeted results (outputs) without contributing to achieving sector purposes and goals. Therefore, project design and implementation without proper attention to purposes and goals (and the verification indicators that allow them to measure the contribution of the project outputs to changes and impacts at a higher level) tend to fall into the situation of “crowding out”, that is, multiple projects competing for already-scarce resources (i.e., labor/workforce time, existing facility, hardware system, and so on). Such phenomenon of micro–macro paradox has been observed and studied in many economic sectors, including health^21^. That is, individual projects showing success: outputs produced as planned within the budget and timeline—but overall, there are no substantial changes toward the desired outcome—such as population health improvement, improved patient experience, etc.

It has been shown that using a systematic approach to project-cycle management that is tightly linked to sector purposes and goals and within sector-wide approach framework can result in desirable outcomes in the medium- and long term (i.e., sustainable)^19,20,22–24^. While the sector-wide approach was developed specifically in relation to development practices, the concept (and its implementation tools) is translatable to other settings/practices. That is, all stakeholders within a sector (funders, lenders, providers, government agencies, and users) implement projects that collectively contribute to achieve the desirable sector goals and purposes, in order to harmonize the efforts, avoid overlapping and wastage, and more importantly magnify each other’s outputs or results. In the case of digital health, it is essential that the EMR implementation, within an organization or settings (hospital, acute care, primary or aged care), starts with the healthcare aims (sector goals) and purposes (operationalized goals).

While the review scope was limited by its focus, i.e., EMR implementation in hospital only rather than EMR/EHR for the whole healthcare system, the diversity of hospital-specific settings, research questions, and methodological analyses posed a challenge to synthesize and present all the information that might be of use for decision-makers and hospital managers. Additionally, the grouping of study types as well as impact domains is limited to our interpretation of the information presented in the original studies. Last, there might be studies qualified to be included, yet missed, in the process of review. A future update and additional in-depth examinations of specific themes identified in this review would undoubtedly progress the research frontier in EMR implementation and economic evaluation.

## Conclusion

Electronic medical records and digital hospitals are becoming a crucial component of healthcare infrastructure and delivery. This trend is likely to accelerate as the technology improves, and digital and artificial intelligent-based technologies become more precise and sophisticated. More investments will be diverted toward the EMR implementation. Yet, an essential part of a valid and reliable business case—economic feasibility studies—remains underdeveloped, as shown in our comprehensive scoping review. Current literature demonstrates a lack of an appropriate economic framework to understand the aims and value of the digital hospital implementation; studies only managed to identify and measure minor benefits and cost. Business cases resting on those narrow analyses, understandably, are failing to gain traction in jurisdictions that still consider EMR rollouts under financial and economic constraints. Further to these concerns is the common observation that disbenefits occur in the short term after implementation; however, such concerns are short-sighted and akin to avoiding a new gym-exercise routine in fear of the inevitable muscle soreness in the days following the first sessions.

## Supplementary information

Supplementary information

## Data Availability

The data generated during the scoping review are available from the corresponding author on reasonable request. However, the majority of such data has been presented in the paper in tables, figures, and text.
