# Supplementary material for: Economic evaluation and analyses of hospital-based electronic medical records (EMRs): a scoping review of international literature
Source: NPJ Digit Med. 2022 Mar 8;5:29. doi: 10.1038/s41746-022-00565-1 (PMC8904550; doi:10.1038/s41746-022-00565-1)
Supplement: Supplementary file 1 — Supplementary information [file 41746_2022_565_MOESM1_ESM.pdf]

SUPPLEMENTARY INFORMATION

Supplementary Table 1: Search terms and results

Databases	Search terms	Results
**Econlit**	digital hospital<br>OR<br>electronic medical record*<br>OR<br>electronic health record*<br>OR<br>emr<br>OR<br>ehr	199
**MEDLINE**	<u>(#1 search)</u><br><br>(MH "Costs and Cost Analysis") OR (MH "Cost-Benefit Analysis") OR (MH "Technology, High-Cost") OR (MH "Health Care Costs") OR (MH "Direct Service Costs") OR (MH "Hospital Costs")<br>OR<br>(AB economic evaluation or cost economic or economic analysis or cost-effectiveness or cost-benefit or cost-consequence or cost-utility)<br>OR<br>(MH "Models, Economic")	273,430
	<u>(#2 search)</u><br><br>“Digital hospital” OR (MH “Electronic Health Records”) OR electronic health record*<br>OR electronic medical record* OR emr OR her	50,855
	#1 AND #2<br>Limit to (English language and humans and yr=”2010-2021”)	1,007

Supplementary References: List of included studies

1. Choi, Jong Soo, Woo Baik Lee, and Poong-Lyul Rhee. "Cost-Benefit Analysis of Electronic Medical Record System at a Tertiary Care Hospital." Healthcare Informatics Research 19, no. 3 (September 2013): 205–14. https://doi.org/10.4258/hir.2013.19.3.205.
2. Li, Kai, Shinji Naganawa, Kai Wang, Ping Li, Ken Kato, Xiu Li, Jie Zhang, and Kazunobu Yamauchi. "Study of the Cost-Benefit Analysis of Electronic Medical Record Systems in General Hospital in China." Journal of Medical Systems 36, no. 5 (2012): 3283–91. https://doi.org/10.1007/s10916-011-9819-6.
3. Beresniak, Ariel, Andreas Schmidt, Johann Proeve, Elena Bolanos, Neelam Patel, Nadir Ammour, Mats Sundgren, et al. "Cost-Benefit Assessment of Using Electronic Health Records Data for Clinical Research versus Current Practices: Contribution of the Electronic Health Records for Clinical Research (EHR4CR) European Project." Contemporary Clinical Trials 46 (2016): 85–91. https://doi.org/10.1016/j.cct.2015.11.011.
4. Iturrate, Eduardo, Lindsay Jubelt, Frank Volpicelli, and Katherine Hochman. "Optimize Your Electronic Medical Record to Increase Value: Reducing Laboratory Overutilization." The American Journal of Medicine 129, no. 2 (2016): 215–20. https://doi.org/10.1016/j.amjmed.2015.09.009.
5. Atasoy, Hilal, Pei-yu Chen, and Kartik Ganju. "The Spillover Effects of Health IT Investments on Regional Healthcare Costs." Management Science 64, no. 6 (2018): 2515–34.
6. Dranove, David, Chris Forman, Avi Goldfarb, and Shane Greenstein. "The Trillion Dollar Conundrum: Complementarities and Health Information Technology." American Economic Journal: Economic Policy 6, no. 4 (2014): 239–70.
7. Driessen, Julia, Marco Cioffi, Noor Alide, Zach Landis-Lewis, Gervase Gamadzi, Oliver Jintha Gadabu, and Gerald Douglas. "Modeling Return on Investment for an Electronic Medical Record System in Lilongwe, Malawi." Journal of the American Medical Informatics Association : JAMIA 20, no. 4 (2013): 743–48. https://doi.org/10.1136/amiajnl-2012-001242.
8. Dupont, Danielle, Ariel Beresniak, Mats Sundgren, Andreas Schmidt, John Ainsworth, Pascal Coorevits, Dipak Kalra, Marc Dewispelaere, and Georges De Moor. "Business Analysis for a Sustainable, Multi-Stakeholder Ecosystem for Leveraging the Electronic Health Records for Clinical Research (EHR4CR) Platform in Europe." International Journal of Medical Informatics 97 (2017): 341–52. https://doi.org/10.1016/j.ijmedinf.2016.11.003.
9. Furukawa, Michael F, T S Raghu, and Benjamin B M Shao. "Electronic Medical Records and Cost Efficiency in Hospital Medical-Surgical Units." Inquiry : A Journal of Medical Care Organization, Provision and Financing 47, no. 2 (2010): 110–23.
10. Kazley, Abby Swanson, Annie N Simpson, Kit N Simpson, and Ron Teufel. "Association of Electronic Health Records with Cost Savings in a National Sample." The American Journal of Managed Care 20, no. 6 (2014): e183–90.
11. Nuckols, Teryl K, Steven M Asch, Vaspaan Patel, Emmett Keeler, Laura Anderson, Melinda B Buntin, and José J Escarce. "Implementing Computerized Provider Order Entry in Acute Care Hospitals in the United States Could Generate Substantial Savings to Society." Joint Commission Journal on Quality and Patient Safety 41, no. 8 (2015): 341–50.
12. Sevick, Laura K, Maria-Jose Santana, William A Ghali, and Fiona Clement. "Prospective Economic Evaluation of an Electronic Discharge Communication Tool: Analysis of a Randomised Controlled Trial." BMJ Open 7, no. 12 (2017): e019139. https://doi.org/10.1136/bmjopen-2017-019139.
13. Spaulding, Trent J, and T S Raghu. "Impact of CPOE Usage on Medication Management Process Costs and Quality Outcomes." Inquiry : A Journal of Medical Care Organization, Provision and Financing 50, no. 3 (2013): 229–47. https://doi.org/10.1177/0046958013519303.
14. Teufel, Ronald J, 2nd, Abby Swanson Kazley, Myla D Ebeling, and William T Basco Jr. "Hospital Electronic Medical Record Use and Cost of Inpatient Pediatric Care." Academic Pediatrics 12, no. 5 (2012): 429–35. https://doi.org/10.1016/j.acap.2012.06.004.
15. Ben-Assuli, Ofir, Amitai Ziv, Doron Sagi, Avinoah Ironi, and Moshe Leshno. "Cost-Effectiveness Evaluation of EHR: Simulation of an Abdominal Aortic Aneurysm in the Emergency Department." Journal of Medical Systems 40, no. 6 (2016): 141. https://doi.org/10.1007/s10916-016-0502-9.

16. DesRoches, Catherine M, Eric G Campbell, Christine Vogeli, Jie Zheng, Sowmya R Rao, Alexandra E Shields, Karen Donelan, Sara Rosenbaum, Steffanie J Bristol, and Ashish K Jha. “Electronic Health Records’ Limited Successes Suggest More Targeted Uses.” Health Affairs (Project Hope) 29, no. 4 (2010): 639–46. https://doi.org/10.1377/hlthaff.2009.1086.

17. Gowrisankaran, Gautam, Keith A. Joiner, and Jianjing Lin. “Does Health IT Adoption Lead to Better Information or Worse Incentives?,” 2016. http://search.ebscohost.com/login.aspx?direct=true&db=ecn&AN=1629399&site=ehost-livehttp://www.nber.org/papers/w22873.pdf.

18. Eastaugh, Steven R. “Health Information Technology Impact on Productivity.” Journal of Health Care Finance 39, no. 2 (2012): 64–81.

19. Encinosa, William E, and Jaeyong Bae. “Will Meaningful Use Electronic Medical Records Reduce Hospital Costs?” The American Journal of Managed Care 19, no. 10 Spec No (2013): eSP19–25.

20. Encinosa, William E, and Jaeyong Bae. “Health Information Technology and Its Effects on Hospital Costs, Outcomes, and Patient Safety.” Inquiry : A Journal of Medical Care Organization, Provision and Financing 48, no. 4 (2012): 288–303.

21. Freedman, Seth, Haizhen Lin, and Jeffrey Prince. “Information Technology and Patient Health: Analyzing Outcomes, Populations, and Mechanisms.” American Journal of Health Economics 4, no. 1 (2018): 51–79.

22. Haque, Rezwan. “Organizational Innovation in Health Care.” Harvard University, 2015. http://search.ebscohost.com/login.aspx?direct=true&db=ecn&AN=1537499&site=ehost-live.

23. Himmelstein, David U, Adam Wright, and Steffie Woolhandler. “Hospital Computing and the Costs and Quality of Care: A National Study.” The American Journal of Medicine 123, no. 1 (2010): 40–46. https://doi.org/10.1016/j.amjmed.2009.09.004.

24. Hydari, Muhammad Zia, Rahul Telang, and William M. Marella. “Saving Patient Ryan--Can Advanced Electronic Medical Records Make Patient Care Safer?” Management Science 65, no. 5 (2019): 2041–59.

25. Miller, Amalia R, and Catherine E Tucker. “Can Health Care Information Technology Save Babies?” The Journal of Political Economy 119, no. 2 (2011): 289–324.

26. Xue, Yajiong, Huigang Liang, Xiaocheng Wu, Hai Gong, Bin Li, and Yuxia Zhang. “Effects of Electronic Medical Record in a Chinese Hospital: A Time Series Study.” International Journal of Medical Informatics 81, no. 10 (2012): 683–89. https://doi.org/10.1016/j.ijmedinf.2012.05.017.

27. Zhivan, Natalia A, and Mark L Diana. “U.S. Hospital Efficiency and Adoption of Health Information Technology.” Health Care Management Science 15, no. 1 (2012): 37–47. https://doi.org/10.1007/s10729-011-9179-2.

28. Zlabek, Jonathan A, Jared W Wickus, and Michelle A Mathiason. “Early Cost and Safety Benefits of an Inpatient Electronic Health Record.” Journal of the American Medical Informatics Association : JAMIA 18, no. 2 (2011): 169–72. https://doi.org/10.1136/jamia.2010.007229.

Supplementary Table 2: Preferred Reporting Items for Systematic reviews and Meta-Analyses extension for Scoping Reviews (PRISMA-ScR) Checklist

SECTION	ITEM	PRISMA-ScR CHECKLIST ITEM	REPORTED ON PAGE #
TITLE			
Title	1	Identify the report as a scoping review.	1
ABSTRACT			
Structured summary	2	Provide a structured summary that includes (as applicable): background, objectives, eligibility criteria, sources of evidence, charting methods, results, and conclusions that relate to the review questions and objectives.	1
INTRODUCTION			
Rationale	3	Describe the rationale for the review in the context of what is already known. Explain why the review questions/objectives lend themselves to a scoping review approach.	2-4
Objectives	4	Provide an explicit statement of the questions and objectives being addressed with reference to their key elements (e.g., population or participants, concepts, and context) or other relevant key elements used to conceptualize the review questions and/or objectives.	2-4
METHODS			
Protocol and registration	5	Indicate whether a review protocol exists; state if and where it can be accessed (e.g., a Web address); and if available, provide registration information, including the registration number.	N/A
Eligibility criteria	6	Specify characteristics of the sources of evidence used as eligibility criteria (e.g., years considered, language, and publication status), and provide a rationale.	6
Information sources*	7	Describe all information sources in the search (e.g., databases with dates of coverage and contact with authors to identify additional sources), as well as the date the most recent search was executed.	6
Search	8	Present the full electronic search strategy for at least 1 database, including any limits used, such that it could be repeated.	Supplementary Table 1
Selection of sources of evidence†	9	State the process for selecting sources of evidence (i.e., screening and eligibility) included in the scoping review.	6-7
Data charting process‡	10	Describe the methods of charting data from the included sources of evidence (e.g., calibrated forms or forms that have been tested by the team before their use, and whether data charting was done independently or in duplicate) and any processes for obtaining and confirming data from investigators.	7-8
Data items	11	List and define all variables for which data were sought and any assumptions and simplifications made.	7-8
Critical appraisal of individual sources of evidence	12	If done, provide a rationale for conducting a critical appraisal of included sources of evidence; describe	N/A

SECTION	ITEM	PRISMA-ScR CHECKLIST ITEM	REPORTED ON PAGE #
		the methods used and how this information was used in any data synthesis (if appropriate).	
Synthesis of results	13	Describe the methods of handling and summarizing the data that were charted.	7-8
RESULTS			
Selection of sources of evidence	14	Give numbers of sources of evidence screened, assessed for eligibility, and included in the review, with reasons for exclusions at each stage, ideally using a flow diagram.	8
Characteristics of sources of evidence	15	For each source of evidence, present characteristics for which data were charted and provide the citations.	Tables 1-3 Studies ID cited in the text
Critical appraisal within sources of evidence	16	If done, present data on critical appraisal of included sources of evidence (see item 12).	N/A
Results of individual sources of evidence	17	For each included source of evidence, present the relevant data that were charted that relate to the review questions and objectives.	Studies ID cited in the text
Synthesis of results	18	Summarize and/or present the charting results as they relate to the review questions and objectives.	Tables 1-3
DISCUSSION			
Summary of evidence	19	Summarize the main results (including an overview of concepts, themes, and types of evidence available), link to the review questions and objectives, and consider the relevance to key groups.	12-21
Limitations	20	Discuss the limitations of the scoping review process.	13-15
Conclusions	21	Provide a general interpretation of the results with respect to the review questions and objectives, as well as potential implications and/or next steps.	21
FUNDING			
Funding	22	Describe sources of funding for the included sources of evidence, as well as sources of funding for the scoping review. Describe the role of the funders of the scoping review.	Title page

JBI = Joanna Briggs Institute; PRISMA-ScR = Preferred Reporting Items for Systematic reviews and Meta-Analyses extension for Scoping Reviews. From: Tricco AC, Lillie E, Zarin W, O'Brien KK, Colquhoun H, Levac D, et al. PRISMA Extension for Scoping Reviews (PRISMAScR): Checklist and Explanation. Ann Intern Med. 2018;169:467–473. doi: 10.7326/M18-0850.
